# Subjective Perceptions of South Korean Parents Regarding the Effectiveness of Taekwondo Education for Adolescents and Its Characteristics: The Q Methodology Application

**DOI:** 10.3390/ijerph18189687

**Published:** 2021-09-14

**Authors:** Wonjae Jeon, Seunghyun Jang, Kihong Joung

**Affiliations:** 1 Department of Senior Sports Course, Daegu Haany University, 1 Hanuidae-ro, Gyeongsan-si 38610, Korea; dreamj007@hanmail.net; 2 Department of Physical Education, Pusan National University, Busandaehak-ro 63beon-gil, Geumjeong-gu, Busan 46241, Korea; 3 Department of Physical Education, Kangnam University, 40 Gangnam-ro, Giheung-gu, Yongin-si 16979, Korea

**Keywords:** Taekwondo, adolescent, adolescence, Q methodology, subjectivity

## Abstract

This study aims to determine why Korean parents provide adolescent children with continuous physical education through Taekwondo. The Q methodology was applied. The final 25 Q-samples were selected by composing the Q-population. Twenty parents who provided their children with Taekwondo education for more than 10 years were designated as the P-sample. Q-sorting was performed on the P-sample. Centroid factor analysis and varimax rotation were performed using version 2.35 of PQ method program. The study observed four factors with a total explanatory variance of 69%. Types 1 to 4 (N = 5, 7, 5, and 3) pertained to a powerful means of enhancing mental health, the driving force behind stable school life and social development, improvement in psychological and social areas for a successful transition to adulthood, and increased awareness of the values of Taekwondo and importance of physical activity, with eigenvalues of 4.59, 6.42, 3.16, and 1.18 and explanatory variances of 0.16, 0.32, 0.12, and 0.09, respectively. Furthermore, consensus statements for each type were investigated as Q18 and Q17. These findings supported the academic foundation of proper Taekwondo education in adolescence and confirmed it as a powerful means of exerting a positive impact on adulthood.

## 1. Introduction

Taekwondo was adopted as a demonstration sports at the 1988 Seoul Olympics and 1992 Barcelona Olympics and as an Olympic medal event at the 2000 Sydney Olympics. Currently, it is considered a major event in the Summer Olympics [1]. In the case of the United States, several elementary schools have adopted Taekwondo as a part of the curriculum for physical education [2]. Moreover, several Taekwondo clubs exist in many universities around the world, including Korea [3].

Taekwondo has existed as a sport with origins in Korea [4]. However, as its educational value began to emerge, it has transformed into one of the world’s most popular sports [5]. Furthermore, World Taekwondo (WT) reported 80 million trainees in 203 countries [6].

Various studies pointed to the educational effects of Taekwondo. For example, Law [7] argued that the objective of Taekwondo is to cultivate character through training. It is known as an educationally effective martial arts for children and adolescents worldwide. Moreover, Akehurst et al. [4] asserted that traditional Taekwondo provides students with many benefits, such as confidence, leadership skills, self-training, self-motivation, respect, and physical fitness as well as the advantages of a sense of achievement, self-defense, and learning motivation. In particular, the authors emphasized that Taekwondo is extremely helpful for students who are unfamiliar with team sports or those who are isolated. Roh et al. [8] argued that Taekwondo did not exert a significant impact on physical strength, cardiopulmonary endurance, strength, flexibility, and cognitive function. However, it significant influenced the emotional and social skills of students in multicultural families. Moreover, Lakes and Hoyt [9] claimed that Taekwondo not only exerted a positive impact on cognition, emotion, self-discipline, and classroom and social behaviors but also improved self-control, which is the most essential aspect of martial arts. Lastly, Toskovic [10] explained that participating in Taekwondo can improve symptoms of tension, depression, and anger.

The abovementioned studies provided evidence of the common educational effects of Taekwondo, such as sociality, confidence, leadership, and self-control. Alternatively, they also displayed slightly different views on specific effects. In other words, research on the educational effects of Taekwondo is continually required.

Scholars reported that the number of children that participate in Taekwondo is increasing with the increase of educational interest in Taekwondo worldwide [11,12]. This phenomenon is more conspicuous in Korea, the mother country of Taekwondo. One can easily find that the majority of children or adolescents frequent Taekwondo gyms in Korea. According to the statistics of the Ministry of Culture, Sports, and Tourism, out of 840,000 Taekwondo trainees in Korea, youths account for 80% (700,000) [13]. In other words, Korean adolescent are educated in Taekwondo gyms from a very young age regardless of gender.

This educational atmosphere in Korea proves that parents have faith and confidence in the effects of Taekwondo training. In addition, the fact that children start Taekwondo at an early age indicates that parents play a significant role in the participation of children in Taekwondo.

Kim and Kim [14] demonstrated that the children’s choice of a Taekwondo gym and participation in Taekwondo can more greatly influence the parents’ educational goals than the children’s will. Kim [15] revealed that the factor of parental involvement in re-enrolling in a Taekwondo gym exerts a moderating effect. In other words, parents have played an important role in the Taekwondo education of their children and laid the foundation for Taekwondo education in Korea as a whole [16]. Hence, these findings indicate that determining the parents’ perceptions is a crucial process in understanding Taekwondo education in Korea.

Nevertheless, an aspect remains unexplained. The majority of studies focused only on the causal relationship between variables, such as service quality, parental satisfaction, persistence intention, and re-enrollment intention. In fact, the fundamental importance of the parents’ perception and characteristics (i.e., tangible awareness) with regard to the effectiveness of Taekwondo education lacks investigation [17,18,19,20,21]. In contrast to the deductive approach for verifying the theoretical hypotheses adopted by prior studies, the current study aims to examine the effectiveness of Taekwondo education as recognized by and from the subjective point of view of parents. Moreover, we focus on categorizing their psychological subjectivity and on identifying the characteristics of each type using the Q methodology. The study aims to determine the reasons that underlie the involvement of Korean parents in their children’s Taekwondo training during adolescence. In this regard, the Q methodology is useful for the objective measurement of the subjective responses of people related to the research topic to shape perception structure and identify characteristics [22].

Thus, this study intends to derive the subjective perceptions of Korean parents regarding their children’s Taekwondo education through the Q methodology and to present various academic discussions. Based on these discussions, the study endeavors to pinpoint important factors to enhance the understanding of the culture of Taekwondo education in Korea.

The study presents the following research questions: What is the subjective perception of Korean parents about Taekwondo education for adolescent children? What are the characteristics and implications of each factor (type)?

## 2. Methods

The Q methodology is useful for investigating human-aware subjectivity and is appropriate for research that aims to reveal personal experiences, preferences, values, and beliefs about various topics [23]. In addition, in contrast to the R methodology, the Q methodology is less of an operational definition of the researcher but more of an operant methodology that represents the perception of participants through their thoughts and language [22]. Therefore, it is an appropriate method for exploring the perceptions of Korean parents regarding the effects of Taekwondo education on the adolescent years of their children.

### 2.1. Q-Population (Concourse of Statements)

The Q-population is a collection of all possible statements (i.e., concourse) on the subject of the study. These statements are collected through literature analysis, group interviews, and in-depth interviews [24]. Therefore, this study constructed the Q-population through a three-dimensional method. First, we conducted a focus-group interview (FGI) with parents who enrolled their children in Taekwondo education at a private educational institution for approximately 10 years, a university professor who majored in Taekwondo, and PhDs who majored in sports pedagogy [25]. Second, we conducted in-depth interviews with each parent [26]. The FGI and in-depth interviews lasted for approximately 60–80 min and was conducted twice. Third, a review of the literature and documents related to the effects of Taekwondo education was carried out [25]. Through this process, we compiled a total of 52 statements (the concourse).

### 2.2. Q-Set (Q-Sample)

The statements were sorted and thematically grouped. Specifically, repetitive statements were removed, whereas confusing statements were reworded for clarity. The statements were then peer reviewed by colleagues unrelated to the project to verify clarity and conciseness and to identify any potential themes that may have been overlooked [25]. Finally, the 25 most representative statements were selected. The rationale for selecting only 25 statements is that if a complex thought process is required, then adopting 30 or less statements is sufficient. In addition, reliability tends to decrease with the increase in the number of Q-samples [27]. Table 1 provides the results. In addition, a reliability test was conducted by performing Q-sorting twice for three respondents. The result was derived through SPSS Statistics 22.0 with *r* = 0.79, which indicated sufficient reliability [28].

### 2.3. P-Set (P-Sample)

Scholars recommend that the P-sample (participants) is selected according to their interest, known views, and expertise in relation to the research topic [29]. Large sample sizes are not required; thus, the P-sample should be strategically sampled to ensure that all potential viewpoints are covered [25]. For statistical reasons, the number of participants should be less than the number of items in the Q-sample [30]. The reason behind this notion is that the characteristics of the population are not inferred from the characteristics of the P-sample [22]. Thus, the P-sample was compiled by the purposeful sampling [31] technique. It is a useful method for selection of information-rich cases for the most effective use of limited resources. This involves identifying and selecting individuals or groups of individuals that are especially knowledgeable about or experienced with a phenomenon of interest [32]. Against this background, we included 20 Korean parents with children with Taekwondo education for approximately 10 years. Table 2 provides the detailed characteristics and factor weights of the P-sample.

### 2.4. Q-Sorting

**Figure 1:**
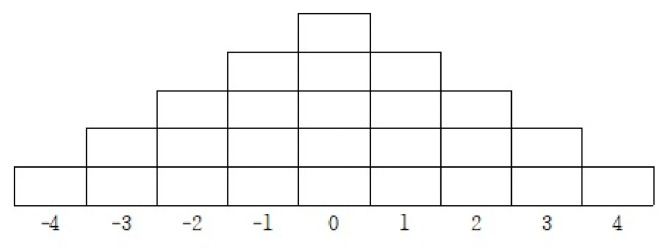
Q−sorting response grid.

Q-sorting denotes that the P-sample preferentially evaluates a previously selected Q-sample [33]. Specifically, after the respondents (P-sample) read and understand the Q-samples, items are rated and arranged in the Q-sorting table (Figure 1), which uses a nine-point scale ranging from *completely agree* (+4) to *completely disagree* (−4). The reason for this rating is because the Q-statement consists of 40 or less items [29]. The Q-sorting method is divided into a forced and an unforced sorting method. Among them, the study employed the forced sorting method. Specifically, the researcher sets the number of responses in the Q-sorting table and instructs the respondents to classify them [29]. This method requires the use of a response grid (Figure 1).

First, the parent participants were asked to read a total of 25 statements and classify them into three groups, namely agree (+), neutral (0), and disagree (−). Those that mostly agreed were classified in order from the right (+4), whereas the items that did not match the most were sorted from the left (−4). The remainder of the statements was considered neutral. Finally, the parents were asked to describe the reasons for selecting the statements grouped as the most positive (+4) and the most negative (−4).

Q-sorting was conducted through an online platform (ZOOM) due to COVID-19 restrictions, and the resulting table was delivered via email. Figure 1 illustrates the Q-sorting response grid.

After categorizing the Q-sorting responses, data were manually input into the software program (PQ Method version 2.35) by entering the statements included for each category from smallest to largest (−4, −3, −2, −1, 0, 1, 2, 3, and 4).

### 2.5. Factor Analysis

A dedicated Q package, that is, version 2.35 of PQ Method [34], was used for factor analysis. The centroid method (i.e., using the orthogonal varimax procedure) was adopted [35]. For factor analysis (a technique that reduces a large number of variables into a smaller number of categories or factors), we identified the most suitable number of factors by entering the number of factors from 7 to 2, and factors with eigenvalues (EVs) of 1.00 or higher were extracted [28].

## 3. Results

### 3.1. Eigenvalue, Variance, and Correlations between Factors (Types)

A total of four types were extracted from the subjectivity study of Korean parents on the effect of Taekwondo education. The EVs for each type are 4.59, 6.42, 3.16, and 1.18, and variance ratios were derived as 0.16, 0.32, 0.12, and 0.09, respectively. The total variance was 0.69, which resulted in an explanatory power of 69% for all types. Moreover, all factors met the Kaiser–Guttman criterion [36]. Table 3 provides the results.

Table 4 demonstrates the correlation between the four types. A close look indicates that the correlation results are 0.26 for Types 1 and 2, 0.51 for Types 1 and 3, and 0.33 for Types 1 and 4. In addition, correlations were derived as 0.42 for Type 2 and 3, 0.05 for Types 2 and 4, and 0.12 for Types 3 and 4.

### 3.2. Type 1 (Factor 1): A Powerful Means of Enhancing Physical and Mental Health

Table 5 presents the results and Z-scores of the statements belonging to Type 1 that were positively or negatively recognized by the participants. In this type, the statements that participants positively agreed with are Q6, Q10, Q8, and Q7, with z-values of 1.78, 1.59, 1.48, and 1.45, respectively. The participants also had the most negative viewpoints for Q1 (Z-score = −2.02).

A total of five participants belonged to Type 1. The P-sample number and the factor weight were P2 (0.5871), P9 (0.4965), P11 (0.6002), P12 (0.6485), and P19 (0.6547). In particular, the results indicate that participant P11 displayed the highest factor weight, which well represents this point of view.

### 3.3. Type 2: The Driving Force behind Stable School Life and Sociability Development

Table 5 shows that the most positive statement of respondents in Type 2 was Q20 (Z-score = 2.01), followed by Q21 (Z-score = 1.69) and Q24 (Z-score = 1.33). In addition, the most negative statement was Q6 (Z-score = −2.00), followed by Q3 (Z-score = −1.71) and Q7 (Z-score = −1.25).

A total of seven participants belonged to Type 2, and this was the largest number of respondents. Respondents P1, P3, P4, P10, P16, P17, and P20 belonged to Type 2, with factor weights of 0.6532, 0.8745, 0.6854, 0.7215, 0.9008, 0.8551, and 0.9221, respectively. The factor weight of participant P20 was the highest, which is a representation of this type of perspective.

### 3.4. Type 3 (Factor 3): Improvement in Psychological and Social Areas Leading to a Successful Transition to Adulthood

In Type 3, the most positive statements were Q11 (Z-score = 2.15), followed by Q13 (Z-score = 1.60) and Q14 (Z-score = 1.16). Furthermore, the most negative statement was Q25 (Z-score = −2.12). Table 5 displays the Q-statements and the Z-scores.

Five participations belonged to this type, and all respondents were strongly aware of the advantages of Taekwondo education for a successful adulthood. Participants P5, P7, P8, P13, and P14 belonged to Type 3, with factor weights of 0.5212, 0.4895, 0.6985, 0.8658, and 0.9745, respectively.

### 3.5. Type 4 (Factor 4): Raising Awareness of the Values of Taekwondo and the Importance of Physical Activity

Table 5 illustrates the positive and negative statements for Type 4 and their Z-scores. The most positively statement was Q5 (Z-score = 2.05), whereas the most negative statement was Q22 (Z-score = −2.16).

Three respondents were identified under Type 4, namely participants P6, P15, and P18, with factor weight of 0.5897, 0.5947, and 0.7098, respectively.

### 3.6. Consensus Statements by All Types

Table 6 indicates that the commonly agreed statements for all types were Q18 (Z-score = 0.78) and Q17 (Z-score = 0.99).

## 4. Discussion

We have discussed the results of the four types and the consensus statements.

In particular, the correlation between Types 1 and 3 was very high, which indicates that Type 1 focuses on improving physical and mental health from adolescence to adulthood, whereas Type 3 recognizes that the advantages of emotional health are necessary factors in adulthood. In other words, the P-sample included in Types 1 and 3 meant that Taekwondo education has a great common perception with regard to the physical and emotional advantages of Taekwondo education, and they commonly recognized Taekwondo training as an essential element for successful adult adaptation.

In contrast, Types 2 and 4 revealed the lowest correlation. Although Type 2 showed a high level of agreement with the advantages of the school life of the youths, respondents under Type 4 agreed more with the value of Taekwondo than with its influence on school life and sociality. These results showed that parents’ perceptions related to Taekwondo education included in each type were clearly separated. Table 2 presents the correlations among the different types. Based on these results, the characteristics of each type are as follows.

First, four respondents belong to Type 1: a powerful means of enhancing mental health. Remarkably, the participants recognize that Taekwondo education is effective in promoting the physical and mental health of their children. Tadesse [37] suggested that the benefits of Taekwondo education for adolescents are multifaceted (i.e., social, benefits, and mental benefits). The descriptions are repeatedly stressed by the other study. The reason underlying this notion is that the achievement of physical and mental health during adolescence is significantly related to adulthood [11]. In fact, Taekwondo training, which regularly occurs during adolescence, creates an opportunity for continuing healthy habits into adulthood [38]. Therefore, the Type 1 results are interpreted as an advantage in the health promotion of Taekwondo education. In addition, the participants agreed relatively positively on the effects of Taekwondo on the children’s mental health. Bing et al. [39] placed a heavy emphasis on the direct relationship between Taekwondo training and mental health. From this perspective, the study views these findings as a confirmation of the perception of the Type 1 respondents.

Second, the largest number of respondents belonged under Type 2. Seven out of 20 P-sample were classified. More specifically, the findings of Type 2 appeared as the strongest perception of participants (P-sample). This type recognized that Taekwondo education exerted a significant impact on their children’s school life. For this reason, the children’s sense of belonging, interest, sociability, and expansion of human relationships within the school became prominent. The reason is that the influence of Taekwondo training exerted a positive effect on adjustment to school life. In other words, students who receive Taekwondo training could establish a wide network of friends in school; thus, they will find school life more enjoyable and interesting [40]. In addition, this type demonstrated beliefs that Taekwondo could be an important means of preventing school violence, which is becoming a social problem in Korea. Choi et al. [41] mentioned that long-term Taekwondo education can reduce school violence behavior in adolescents. In this regard, we find that the Type 2 participants recognize the results of Taekwondo training as significant. Meanwhile, this type of respondents expressed low levels of agreement on the physical and mental benefits of Taekwondo education as well as negative perceptions about the improvement of their children’s Taekwondo-related skills.

Third, Type 3 respondents perceived that taekwondo education was effective in in instilling values that could lead to a successful adulthood. Notably, this Type 3 shows a high correlation with the first type. It means that there are many things in common in the perceptions of parents belonging to the two types. Parents of this type judged that through Taekwondo education, children can learn about thoughtfulness and respect for others as well as gain confidence in various fields. This is because children could also take responsibility for their actions and gain experiences that can improve self-esteem [7]. Consequently, the social support of parents regarding Taekwondo training is an important means of enhancing the patience of the children and instilling a spirit of competition. In other words, it is a useful means of acquiring various personal and social characteristics, which can be carried over into adulthood. Previous studies reported that a high level of emotion regulation in childhood is a robust antecedent of positive social functioning in adolescence [42]. As demonstrated by the Z-score of this type of negative Q-statement in Table 5, the Type 3 participants held negative perceptions about expanding career opportunities through Taekwondo education.

Lastly, the perception of the value of various aspects of Taekwondo was remarkable among the Type 4 respondents. The parents selected their children’s careers as athletes or coaches through long-term Taekwondo education. Parents recognized that Taekwondo education had an effect on their children’s career choice in the near future. For this reason, this type selected in a high Z-score for Q25. Moreover, this group of respondents displayed strong agreement with Q5 and Q9, which indicate that Taekwondo education enabled them to gain healthy eating habits and that it exerted a significant impact on the importance of body care [43]. These findings reveal that healthy body management during adolescence exerts a significant effect on adulthood. Tadesse [44] explained that Taekwondo training can positively contribute to the well-being of adolescents; nevertheless, Type 4 participants exhibited mostly negative perceptions with regard to statements related to school life adaptation in Taekwondo education.

Finally, all respondents positively agreed with Q18 (Z-score = 0.78) and Q17 (Z-score = 0.99). It means that all respondents placed these two statements in a positive place (in Q-sorting response grid). Taekwondo education is widely agreed as being able to change the life of the children in a positive manner. In particular, the respondents mentioned that their children acquired a well-planned lifestyle and built great character in adolescence because sportsmanship and the spirit of fair play could be cultivated through Taekwondo education [45]. This fact could be interpreted as the basis for proving the value of building a personality, a developing lifestyle, and a successful transition for adulthood that can be obtained by participating in Taekwondo education.

## 5. Conclusions

The majority of youths in South Korea participate in Taekwondo training from a very early age, which is due to the philosophy of their parents about education [12]. What values and effects do Korean parents instill in their children after a long-term enrollment in Taekwondo education? To address these questions, the study established the types of subjective perception and the characteristics of parents regarding Taekwondo education. For this purpose, the study employed the Q methodology. The final 25 Q-samples were selected by composing the Q-population. Among the Korean parents, 20 who enrolled their children in Taekwondo education for approximately 10 years were selected as the P-sample. The result pointed to four types, with a total explanatory variance of 69%. Types 1 to 4 pertain to Taekwondo education as a powerful means of enhancing mental health, the driving force behind stable school life and social development, improvement in psychological and social areas leading to a successful transition to adulthood, and raising awareness of the values of Taekwondo and the importance of physical activity, respectively. Furthermore, the consensus statements between each type were identified as Q18 and Q17.

Type 1 indicates that regular Taekwondo training for teenagers is an effective physical activity that may improve physical and mental health. In fact, Taekwondo practice can improve anaerobic or muscle strength [11], which is an important reason to improve their sense of achievement and satisfaction by increasing their physical self-efficacy [46]. Therefore, the results under Type 1 can lead to substantial academic outcomes due to the positive impact of these factors on the well-being of students.

The participants recognized that Taekwondo education exerted a significant impact on their children’s school life and social skills. Elementary school students trained in Taekwondo for a long time tend to display high levels of ego resilience, which can lead to factors that help them adapt to school life and develop social skills [47]. Ego resilience is a dynamic ability to control one’s ability to cope with environmental factors [48]. On this basis, long-term participation in Taekwondo education may be a reason for its important position in the curriculum for physical education and a foundation for the successful adult life of teenagers.

According to the last finding, parents agreed that students may acquire the values of Taekwondo as a sport and the value of physical activity by participating in Taekwondo. In fact, the value of Taekwondo as a martial art occupies an important position in the curriculum for physical education, and the pedagogical practices for Taekwondo could lead to progress among students [49]. Accordingly, the study infers that Korean parents perceived this value, which led to their children’s participation in Taekwondo.

Based on the abovementioned discussion, we conclude that long-term Taekwondo education for young people not only produces various positive factors but also serves as a powerful means to achieve a successful adulthood. Hence, the study hopes that not only Korean parents but also parents around the world should consider their children’s participation in Taekwondo education from infancy to adolescence.

## Figures and Tables

**Table 1:** Q-statements.

Q Number	Q-Statements
1	It can instill knowledge about the historical value of Taekwondo, which is popular worldwide.
2	It can instill knowledge about the social and cultural values of Taekwondo.
3	By polishing Taekwondo techniques and skills, youths can increase their desire for promotion and rank test.
4	It increases interest in sports during adolescence, which the youths may carry into adulthood.
5	It can help children recognize the positive effects of physical activity and the importance of taking care of their bodies.
6	It can improve muscular strength, muscular endurance, flexibility, and cardiovascular endurance, among others, which are essential in adolescence.
7	It can promote agility, speed, and coordination, which are essential not only in adolescence but also in adulthood.
8	It is helpful for managing body weight, preventing obesity, and dieting (weight loss).
9	It leads to healthy eating habits.
10	It can relieve stress and reduce depression.
11	It is one of the important methods of teaching about thoughtfulness and respect for others.
12	It can actively change a child’s personality.
13	It can build confidence in various fields and improve self-esteem.
14	It can inspire a sense of responsibility and achievement for children.
15	It can improve patience and instill a spirit of competition.
16	It enables young people to acquire skills necessary for life safety, such as self-defense.
17	It is one of the significant methods for learning about manners and etiquette.
18	It can positively change the attitude of living at home, and it leads to the acquisition of a planned life habit.
19	It can induce improvement in class concentration and academic performance.
20	It can promote a sense of belonging within the school and make school life more enjoyable and interesting.
21	It could be a means of stimulating and improving friendships.
22	It can foster close relationships with instructors or teachers.
23	It can be a means for guiding, maintaining, and improving social relationships with various individuals.
24	It becomes a medium for preventing bullying and school violence.
25	It provides opportunities for career expansion (professional Taekwondo players or instructors).

**Table 2:** Summary of characteristics of the P-sample and a defining sort.

Factor (Type)	P-Sample	Age	Child’s Age	Gender	Education (Years)	Starting Age	Factor Weight
Type 1 (N = 5)	2	53	19	Female	11	5	0.59
	9	49	18	Male	12	5	0.49
	11	56	17	Male	10	6	0.60
	12	56	16	Female	9	5	0.64
	19	42	15	Female	8	5	0.65
Type 2 (N = 7)	1	53	18	Male	10	7	0.65
	3	48	16	Female	10	6	0.87
	4	47	17	Female	11	5	0.69
	10	46	16	Male	9	5	0.72
	16	46	18	Female	10	7	0.90
	17	50	19	Male	9	5	0.86
	20	50	17	Male	10	5	0.92
Type 3 (N = 5)	5	44	17	Female	10	5	0.52
	7	47	18	Male	11	6	0.49
	8	45	16	Female	10	5	0.70
	13	49	19	Male	13	6	0.86
	14	51	19	Female	12	6	0.97
Type 4 (N = 3)	6	54	19	Female	11	5	0.58
	15	46	17	Male	10	5	0.59
	18	52	18	Male	12	5	0.71

**Table 3:** Eigenvalue (EVs) and variance between types.

	Type 1	Type 2	Type 3	Type 4
Eigenvalue	4.59	6.42	3.16	1.18
% of explanatory variance	0.16	0.32	0.12	0.09
Total variance	0.16	0.48	0.6	0.69

**Table 4:** Correlations between types.

	Type 1	Type 2	Type 3	Type 4
Type 1	1			
Type 2	0.26	1		
Type 3	0.51	0.42	1	
Type 4	0.33	0.05	0.12	1

**Table 5:** Statements with Z-scores of ±1.00 (or higher) type 1 to type 4.

		Q-Statement	Z-Score
Type 1	Positive	6. It can improve muscular strength, muscular endurance, flexibility, and cardiovascular endurance, which are essential in adolescence.	1.78
		10. It relieves stress and reduces depression.	1.59
		8. It is helpful for managing body weight, preventing obesity, and dieting (weight loss).	1.48
		7. It can promote agility, quickness, and coordination, which are essential not only in adolescence but also in adulthood.	1.45
	Negative	2. It can instill knowledge about the social and cultural values of Taekwondo.	−1.84
		1. It can instill knowledge about the historical value of Taekwondo, which is popular around the world.	−2.02
Type 2	Positive	20. It can promote a sense of belonging within the school and make school life more enjoyable and interesting.	2.01
		21. It could be a means of stimulating and improving friendships.	1.69
		24. It becomes a medium for preventing bullying and school violence.	1.33
	Negative	7. It can promote agility, quickness, and coordination, which are essential not only in adolescence but also in adulthood.	−1.25
		3. By polishing Taekwondo techniques and skills, youths can increase their desire for promotion and rank.	−1.71
		6. It can improve muscular strength, muscular endurance, flexibility, and cardiovascular endurance, among others, which are essential in adolescence.	−2.00
Type 3	Positive	11. It is one of the important methods for teaching thoughtfulness and respect for others.	2.15
		13. It can build confidence in various fields and improve self-esteem.	1.6
		14. It can inspire a sense of responsibility and achievement for children.	1.16
	Negative	24. It becomes a medium for preventing bullying and school violence.	−1.53
		1. It can instill knowledge about the historical value of Taekwondo, which is popular worldwide	−1.74
		25. It provides opportunities for career expansion (as professional Taekwondo players or instructors).	−2.12
Type 4	Positive	5. It can help children recognize the positive effects of physical activity and the importance of taking care of their bodies.	2.05
		2. It can instill knowledge about the social and cultural values of Taekwondo.	1.88
		25. It provides opportunities for career expansion (as professional Taekwondo players or instructors).	1.87
		9. It leads to healthy eating habits.	1.74
	Negative	24. It becomes a medium for preventing bullying and school violence.	−1.32
		21. It could be a means of stimulating and improving friendships.	−1.99
		22. It can foster close relationships with instructors or teachers.	−2.16

**Table 6:** Consensus statements.

Q-Statement	Z-Score
18. It can positively change the attitude of living at home, and it leads to the acquisition of a planned life habit.	0.78
17. It is one of the significant methods for learning about manners and etiquette.	0.99

## Data Availability

The data presented in this study are available upon request from the corresponding authors.
